# Space-to-Ground Communication for Columbus: A Quantitative Analysis

**DOI:** 10.1155/2015/308031

**Published:** 2015-07-28

**Authors:** Thomas Uhlig, Thurid Mannel, Antonio Fortunato, Norbert Illmer

**Affiliations:** ^1^Columbus Control Center, Deutsches Zentrum für Luft- und Raumfahrt (DLR), Oberpfaffenhofen, 82234 Wessling, Germany; ^2^European Astronaut Center, Deutsches Zentrum für Luft- und Raumfahrt (DLR), Linder Höhe, 51147 Köln, Germany

## Abstract

The astronauts on board the International Space Station (ISS) are only the most visible part of a much larger team engaged around the clock in the performance of science and technical activities in space. The bulk of such team is scattered around the globe in five major Mission Control Centers (MCCs), as well as in a number of smaller payload operations centres. Communication between the crew in space and the flight controllers at those locations is an essential element and one of the key drivers to efficient space operations. Such communication can be carried out in different forms, depending on available technical assets and the selected operational approach for the activity at hand. This paper focuses on operational voice communication and provides a quantitative overview of the balance achieved in the Columbus program between collaborative space/ground operations and autonomous on-board activity execution. An interpretation of the current situation is provided, together with a description of potential future approaches for deep space exploration missions.

## 1. Introduction

Flight control operations for the International Space Station (ISS) are carried out [1] around the clock by five major Mission Control Centres (MCCs): Mission Control Centre-Houston (MCC-H), located in Houston (United States); the Payload Operations and Integration Centre (POIC), located in Huntsville (United States); the Columbus Control Centre (Col-CC), located near Munich (Germany); the Space Station Integration and Promotion Centre (SSIPC), located in Tsukuba (Japan); and finally the Tsentr Upravleniya Poloyotami (TsUP) located near Moscow (Russia).

Flight controllers at those centres and the crew on board the International Space Station rely primarily on a constellation of geostationary satellites, the Telemetry and Data Relay Satellite System (TDRSS), to carry out their communication. As the International Space Station orbits around the Earth, it transmits radio signals in both S-band (for low data rate telemetry and for telecommands) and Ku-band (for high data rate transmissions) to those satellites, which in turn relay them to either a main ground station in White Sands or to a remote terminal located on the island of Guam. Data is then securely transferred from those facilities to the intended users in the various Mission Control Centres [2].

This communication link is, in theory, permanently available (in practice, since the TDRSS is a shared multiuser system, the ISS only gets a limited number of preplanned communication slots; those slots can be complemented in real time in case of need, i.e., an on-board emergency) and enables a multitude of technical possibilities for information exchange. In the broadest sense, communication can, for example, take place by email exchange, private phone calls, bidirectional video conferences, or direct, semipublic operational voice communication. This latter option, normally carried out over two channels in S-band (S/G 1 and S/G 2) and two channels in Ku-band (S/G 3 and S/G 4), constitutes the primary way to perform official, work related discussions and it represents the main focus of this study.

## 2. Operational Voice Communication

Operational voice communication over the space-to-ground voice channel (S/G) is historically carried out by only one flight control position per Mission Control Centre: the Spacecraft Communicator [3]. In the framework of the International Space Station program, this position assumes different names (or “call signs”) in each of the involved centres: it is called CAPCOM in MCC-H, PAYCOM in POIC, EUROCOM in Col-CC, J-COM in SSIPC, and Glavni in TsUP. These five positions are, under normal circumstances, the “voices” of the Mission Control Centres: they collect all necessary information from the other flight controllers, cross-check it for consistency and appropriateness, package it together in a concise and efficient manner, and finally relay it to the astronauts on board (in special situations, responsibility for space-to-ground communication may fall on the Flight Director). The interaction between the astronauts and Mission Control has been the subject of different studies over the years, also from a psychological perspective [4, 5].

From the point of view of any specific Mission Control Centre, despite the greatly improved technical assets currently available, operational voice communication is generally limited to only very specific, preplanned communication slots. The most important of such opportunities are the Daily Planning Conferences (DPC): two 30 min round calls placed at the beginning (Morning Daily Planning Conference) and at the end (Evening Daily Planning Conference) of the crew working day, where control centres discuss with the astronauts the current schedule, on-board and ground situation, and any critical issue requiring resolution.

Outside these conferences, operational voice communication can be either triggered by specific procedure steps or be altogether unplanned. In the first case, verbal information exchanges are directly included in the procedure to ensure that crew and ground are properly synchronised in the execution of the activity flow. In the second case, unforeseen events or questions happening on either side of the communication link might require a close coordination and therefore a sometimes extensive communication between the two operational groups. Planned as well as unforeseen communications are both discussed and investigated in more detail below.

All operational communications are performed in a very formal manner, which ensures short, clear, and unambiguous information transfer. A voice protocol comparable to the standards in military or aviation is used (see, e.g., [6]). During a conversation with the crew all teams on the ground are required to pause their ongoing operations and, if possible, solely focus on the transmission. This ensures overall situational awareness, quick response times in case of questions, and effective information sharing. All communications are also recorded at the control centers and can be replayed if need be. The operational relevance of verbal communications with the astronauts cannot be overestimated.

## 3. Methodology

The following methodology has been used in all the analyses of crew communications and crew procedures presented in this paper:

1. Only ESA activities related to the Columbus module and its payloads were considered.
2. The selected activities span over a timeframe of more than half a year.
3. This timeframe falls after the end of the assembly phase for the International Space Station.
4. In case of multiple conductions of the same activity (same activity name identifier, same procedure steps), only the first occurrence was included (unless otherwise mentioned) to ensure that in the analysis routine tasks are not outbalancing one-time activities.

The number of samples for each statistic is provided as value *N* in all tables. The investigation of the voice communication content was carried out based on the log book entries of the Flight Control Team, which is required to document all major events during a shift, among them crew calls.

## 4. Crew Procedures

**Figure 1:**
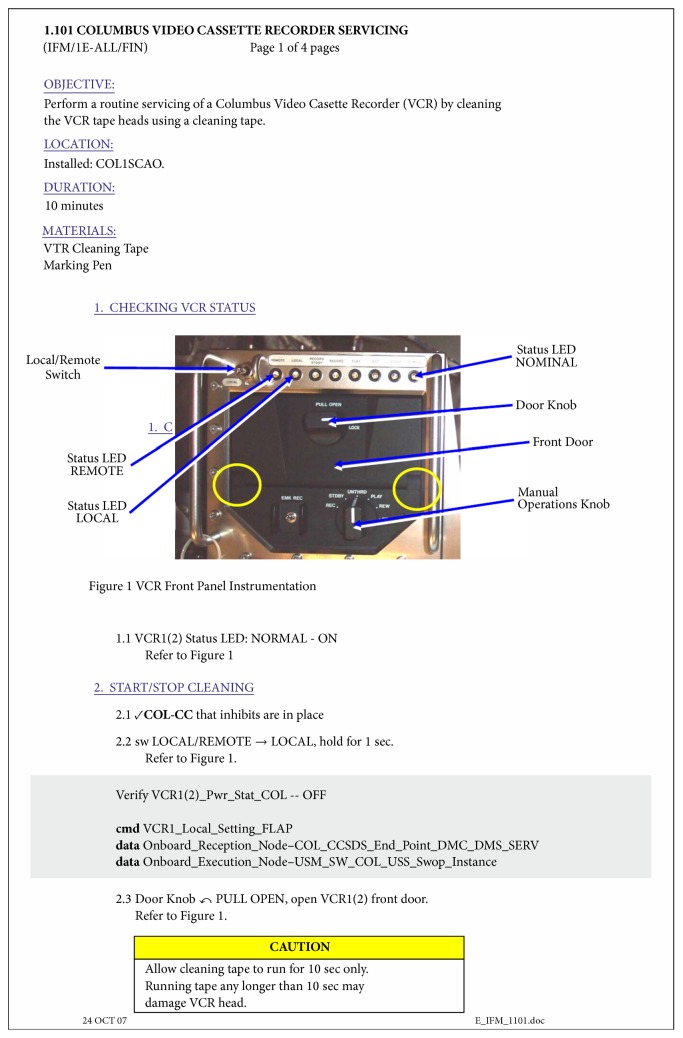
Operations Data File with mandatory check with Col-CC.

Most of the tasks on board the International Space Station are described by a well-defined set of Standard Operating Procedures (SOP), the so-called Operations Data Files (ODF) (Figure 1 or [7]). These are step-by-step instructions designed to allow the crew a certain degree of autonomy while, at the same time, retaining the possibility (in some cases the need) for the Flight Control Teams on the ground to intervene. Operations Data Files follow well-defined rules and syntax [8]. Their content is reviewed by multiple experts and the approval process is overseen by a dedicated control board. They are uplinked to the space station electronically in a dedicated library, the International Procedure Viewer (IPV), which is also replicated on the ground for control centres use.

## 5. Planned Crew Communication

At the time of procedure authoring, a decision must be taken on the amount of desired interaction between crew and ground. Such decision depends in part on the nature of the activity itself—for instance, whether or not it can or must be executed by the astronauts in complete autonomy—and in part on a number of other considerations such as available crew time, expected communication coverage, and amount of necessary ground support. It is often the case, in fact, that multiple parts of a procedure can be executed either by the crew or by ground and a judgment call has to be made based on best estimate of the boundary conditions at execution time. This early decision can, to a certain extent, still be changed at a later stage as described in Section 6.

**Figure 2:**
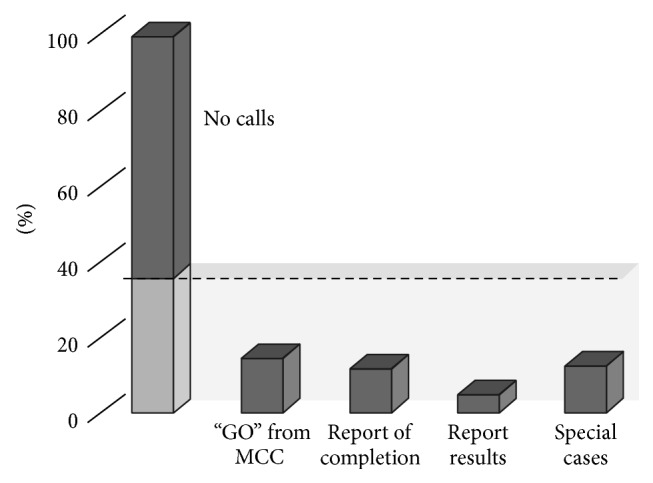
Types of communications used in procedures (*N* = 160).

Figure 2 provides a summary of the type of crew calls included in the analysed procedures.

The analysis shows that 64% of the procedures do not require any space-to-ground interaction: they can be considered as examples of autonomous crew operations. In the remaining 36%, crew communication is foreseen to cover a number of different operational needs (note that the same procedure can contain different types of crew calls; hence, the sum of the percentages provided amounts to more than 100%).

Specifically, 15% of the procedures require the crew to get a dedicated GO before selected steps can be executed. This GO has a pure informational character in two-thirds of the cases (“We want the big picture about what the crew is doing”), whereas the remaining one-third really serves the purpose of a mandatory coordination. In this case, ground has usually an action (check system or facility status, change system configuration by telecommand, etc.) before the crew is given the GO to proceed.

In 12% of the cases a “report completion” is requested at the end of the procedure or after selected steps. This communication serves again two different purposes: 84% of the times this report is just a status, which provides the ground team with a better insight into the current crew schedule. In the remaining 16%, information transfer is crucial because the ground team needs to continue the activity via ground commanding and needs the crew's confirmation of completion as entry point for their part of the procedure.

In 5% of the analysed procedures, the crew is asked to transfer to Mission Control information that cannot be acquired by other means: stowage locations, readings of mechanical devices not connected to the station's data management system, identifiers of used consumables, and so forth.

Finally, 13% of the procedures instruct the crew that, under certain circumstances (“if xxx, then call yyy”), the ground team has to be consulted or informed. In many of those cases crew can follow a “nominal procedure path” without communication with ground, and only a deviation from the “nominal branch” requires a space-to-ground call.

This analysis shows that the majority of the procedures only contain crew communications as “nice to have,” if at all. In the rare cases in which communications are a hard requirement for successful operations it serves either the purpose of coordination between the two acting teams (crew and ground), it transfers information which cannot be gained otherwise, or it was introduced to decrease the level of complexity of the procedure.

Hence in case a minimization of procedurally required communications and therefore maximum crew autonomy is targeted, the following factors need to be considered: the procedure format needs to allow a higher degree of complexity without overwhelming the crew with information. The on-board system needs to provide the capability to have more insight into its status via telemetry or video data. Convoluted operations and dependencies between crew and ground operations need to be avoided or the sync points made available via other means.

## 6. Timeline and Execution Notes

**Figure 3:**
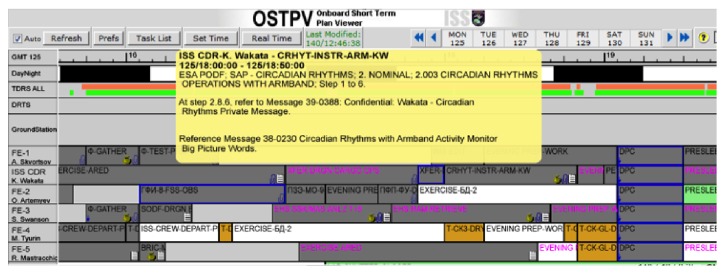
Example of on-board timeline with Execution Note details for an arbitrary activity called up (yellow box).

In real-time operations, the astronauts' tasks for any given day are summarised in the On-Board Short Term Plan (Figure 3), a timeline where the instructions necessary to execute each activity are provided in “Execution Notes.” A proper analysis of operational voice communication cannot be carried out without a brief discussion of Execution Notes and how they are used, as they represent a procedure's entry point into the real implementation context.

Execution Notes come in different types. In the simplest (and rarest) case, they link to a specific procedure that the crew has to execute in its entirety. They can also be used, however, to provide additional instructions, modify the execution flow of a procedure, or directly provide the required actions for very easy tasks rather than establishing a link to a dedicated, more extensive ODF.

**Figure 4:**
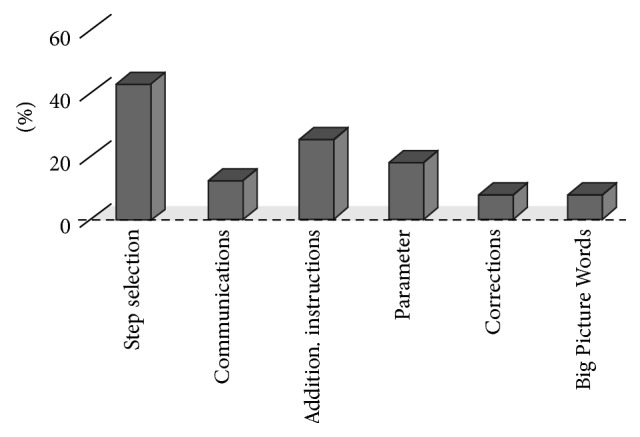
Use of Execution Notes (*N* = 160).

Figure 4 provides a statistical overview of the different uses of Execution Notes.

In 43% of the activities, the Execution Note was used to point the crew only to certain steps of a procedure. This possibility eases the work of the ground teams, which do not need to generate new procedures for each slight variation of the same activity. However, the likelihood of crew mistakes increases with the number of procedure steps which are listed as “to be skipped” or “to be executed” in the Execution Note.

In 12% of the Execution Notes considered, additional requests were added for the crew to tag up with Mission Control. This approach is mostly used when only parts of procedures are executed and steps which would already contain synchronisation points with Mission Control are skipped.

In 25% of the activities under investigation the Execution Note contained instruction replacing a procedure reference. This is especially useful when tasks are simple and can be described in just a few lines of text (in case actions are extracted from a larger Operations Data File, a link to the original document is generally provided as a reference).

In a few cases, procedures may include parameters, data not provided in the procedure itself but included instead in the Execution Note. Typical examples are directory locations or file names, user identifiers, or other variables in science protocols. This philosophy was used in 18% of the activities and is a robust way to specialise a generic procedure with additional information.

In some occasions (8%), Execution Notes were used to correct some procedure details. In case the actual on-board situation is slightly different than the baseline assumed in the procedure, this is a convenient way to adapt without the need for a full-fledged procedure update process. Of course, special care needs to be taken to make sure that the final “product” (procedure + Execution Note) is still valid, especially in its safety relevant parts.

For complex crew operations, it is often beneficial to provide the crew with the big picture: how the corresponding activity fits into the overall scenario or what high-level goals shall be achieved. In these cases, additional documents provide “Big Picture Words” (BPW) for the crew and are linked as reference via Execution Note. This approach was used in 8% of the activities under investigation.

In summary it can be stated that Execution Notes not only are establishing the link between the activities on the timeline, but are sometimes used to convey additional information to the crew. However, this approach increases the likelihood of errors, since the crew has now information about activity execution in two places (ODF and Execution Note). For that reason a new concept is currently discussed to reflect the information from the Execution Note directly in the ODF: procedure steps to be disregarded will not be displayed; comments and additional information for dedicated steps will appear directly at the corresponding location of the procedure. This is not changing the basic concept but ensures a better integration of both means of written communication.

## 7. Real-Time Activity Execution

**Figure 5:**
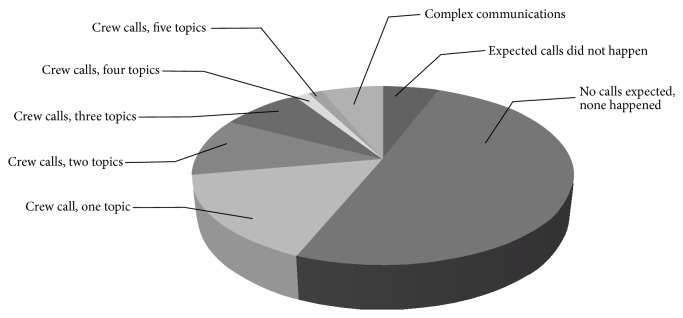
Crew calls statistics (*N* = 144).

A post-execution analysis unveiled that 57% of the activities in orbit were executed without any communications between the control centre and the astronauts: 51% were without communication as planned, whereas 6% required communication according to either procedure or Execution Note, but it did not take place. For the remaining 42% of the activities communications between the control centre and the crew took place, as indicated in Figure 5.

The statistics show that most of the activities required communications for one topic only (16%), while in some cases the need arose to discuss two (10%), three (8%), four (1%), or five (1%) topics.

**Figure 6:**
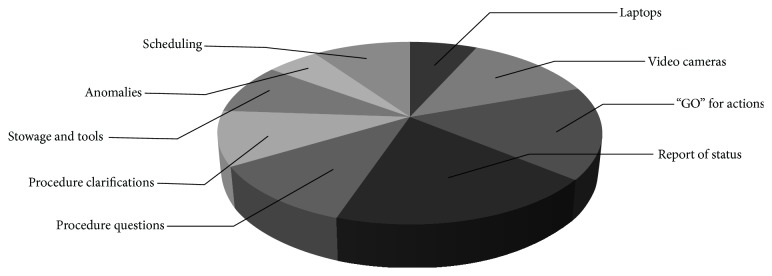
Subjects addressed during crew calls (*N* = 134).

It is interesting to also look at the topics which have been addressed during activity related crew communications. An analysis of this aspect is depicted in Figure 6.

The highest ranking have crew reports of the status (19%), followed by astronaut's requests to get a GO from the Flight Control Team to proceed with certain activity steps (16%). Most of those crew calls were requested by procedure or Execution Notes (“Report to MCC,” “Request GO from MCC”) and served as synchronisation point.

In 13% of the cases, the communication was related to video transmission from the space station to ground. In some cases the field-of-view requirements of the ground team were addressed, but in most cases it was just the coordination for “coming on board” to be addressed: since the International Space Station is not only a science laboratory, but also the private living quarters of the astronauts, strict privacy rules require the MCCs to ask for permission before any sort of video equipment is activated on board (slightly different requirements apply depending on the location on station and the nature of the activity being filmed).

The procedure itself was the subject of discussion in other cases: in 11% crew had questions about the content of the procedure and in 10% it was the MCC, which initiated the discussion, because the on-board configuration required further explanations or even corrections to what had been provided in writing.

10% of the space-to-ground discussions were related to activity scheduling—whether or not they could be anticipated or postponed—and 9% of the activity related communications were attributed to stowage locations or tools.

In 7% of the cases, the crew had issues with the computer hardware or software and in an additional 7% the communications were related to anomalies experienced during activity execution.

It is interesting to point out that the majority of space-to-ground communications are, in a certain sense, predictable—requested by procedure or by a dedicated process. As a consequence, the EUROCOM (the same applies to different extents also for other Spacecraft Communicators) is not required to cover on console the entire crew work day but its shifts are focused on the periods where activities under responsibility of Col-CC take place.

It also becomes apparent that only a small part of the communications cannot be avoided at all—everything related to anomalies and questions of the crew.

## 8. Summary and Outlook

International Space Station operations rely on the availability of dedicated communication assets for the coordination between the astronauts in the space and the Mission Control Centres on the ground. By means of written instructions the amount of real-time communication is minimised and shifted to near-real-time via timeline, Execution Notes, and Operations Data Files.

This paper analysed approximately 1/2 a year of Columbus related voice communication and classified it into different types. 36% of the investigated procedures (ODF) required verbal communication; almost all of this can however be avoided by improved procedure standards, improved on-board systems, or decoupling of crew and ground operations.

However, still a large amount of activities requires real-time communications, which in some cases is not just convenient but rather crucial. This is especially true for all communications linked to off-nominal and unplanned situations.

Future missions with significant communication delays (a trip to Mars could experience delays up to the range of 1/2 an hour) will require significant adaptations of the operations concept, and Mission Control Centres will rather assume the role of Mission Support Centres due to the impossibility of truly real-time communication.

**Figure 7:**
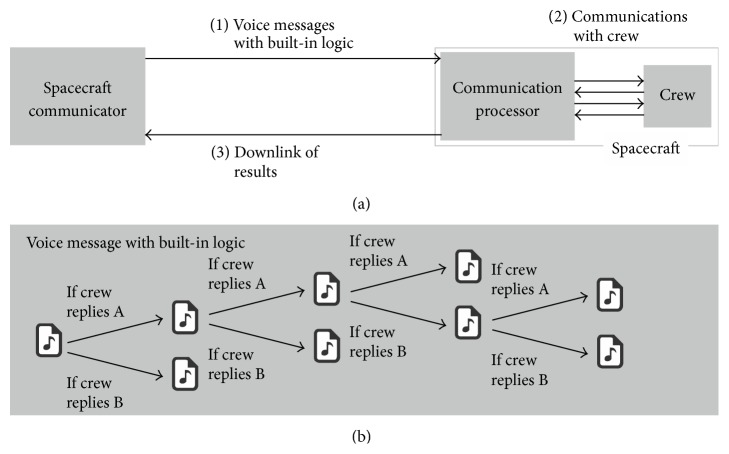
Potential communication approach for future missions.

Innovative communications concepts as well as different team dynamics [9] will need to be devised, and one possible example is shown in Figure 7.

This approach foresees sending not just a single voice message to the remote spacecraft, but rather a multitude of them embedded into a logical structure, which is analysed by the on-board computer and presented to the crew based on their responses. Only when the predefined communication is terminated are the results sent back to the ground, and they can be reviewed by the Flight Control Teams.
